# Prevalence and Risk Factors of Home Quarantine Strategy Implementation Among Chinese Residents During the Coronavirus Disease 2019 Pandemic

**DOI:** 10.3389/fpsyg.2021.679538

**Published:** 2021-09-14

**Authors:** Liqing Li, Xin Shen, Xiaogang Zhou, Hui Cao, Jing Feng, Zihui Lei, Kunming Tian, Jiarui Liang, Yuan Wang, Zuxun Lu, Yong Gan

**Affiliations:** ^1^Department of Management Science and Engineer, School of Economics and Management, Jiangxi Science and Technology Normal University, Nanchang, China; ^2^Department of Social Medicine and Health Management, School of Public Health, Tongji Medical College, Huazhong University of Science and Technology, Wuhan, China; ^3^Department of Human Resource Management, School of Economics and Management, East China Jiaotong University, Nanchang, China; ^4^Department of Labor Economics and Management, Beijing Vocational College of Labour and Social Security, Beijing, China; ^5^Department of Preventive Medicine, School of Public Health, Zunyi Medical University, Zunyi, China; ^6^Department of Preventive Medicine, Institute of Reproductive Health, Tongji Medical College, Huazhong University of Science and Technology, Wuhan, China; ^7^Department of Social Medicine and Health Management, Centre for Health Management and Policy Research, School of Public Health, Cheeloo College of Medicine, Shandong University, Jinan, China; ^8^National Health Center Key Lab of Health Economics and Policy Research (Shandong University), Jinan, China; ^9^Department of Social Medicine and Health Management, School of Management, Shanxi Medical University, Taiyuan, China

**Keywords:** home quarantine strategy, Chinese residents, coronavirus disease 2019, prevalence, risk factors

## Abstract

**Background:** Home quarantine is an important strategy to contain the mass spread of the coronavirus disease 2019 (COVID-19) pandemic. However, there are a dearth of studies on the prevalence and risk factors of home quarantine strategy implementation among residents. This study aims to assess the state of home quarantine strategy implementation among Chinese residents, which could provide a reference for quarantine policymakers around the world during the pandemic.

**Method:** We conducted a cross-sectional survey of 3,398 residents in China by adopting a convenience sampling strategy. We measured the prevalence and risk factors of home quarantine strategy implementation with the Center for Epidemiological Studies-Depression Scale (CES-D), 10-item Connor-Davidson Resilience Scale (CD-RISC 10), and Perceived Social Support Scale (PSSS). A multivariable model was used to determine the factors associated with home quarantine strategy implementation.

**Results:** A total of 2,936 (86.4%) respondents carried out home quarantine. There were some factors significantly associated with home quarantine strategy implementation among Chinese residents during the COVID-19 outbreak. Respondents who were male, lived in western and central China, were aware of the primary symptoms of COVID-19, were willing to accept recommendations on relevant protective measures, understood local quarantine measures, had better resilience, and had better social support were more likely to engage in home quarantine. Respondents who were married, were employed, were healthy, and had high depression scores were more likely to refuse to follow home quarantine guidance.

**Conclusions:** Gender, region, marital status, employment status, health status, awareness of the primary symptoms of COVID-19, willingness to accept recommendations on relevant protective measures, understanding of local quarantine measures, depression, psychological resilience, and perceived social support were the main factors affecting the implementation of residents' home quarantine strategy. Health service policymakers should adopt relevant measures to improve the prevalence of home quarantine strategy implementation among residents during the pandemic.

## Background

Home quarantine is the separation and restriction of movement of people who have potentially been exposed to a contagious disease to limit disease spread (Wang et al., 2020). Particularly during the early stages of a novel infectious disease outbreak, quarantine can be applied to large numbers of people. Home quarantine is necessary and effective for preventing the spread of coronavirus disease 2019 (COVID-19) (Wang and Wang, 2020). China adopted the “Reduce travel and contact with others” policy as the core of the nationwide home quarantine strategy early in the COVID-19 outbreak (Bauch and Anand, 2020). With the global spread of the virus, many countries have issued similar home quarantine policies (Matias et al., 2020). Previous studies reported that the effects of home quarantine on the prevention and control of COVID-19 are specific and remarkable. A study conducted by McCloskey et al. (2020) demonstrated that implementing a home quarantine strategy is an effective alternative to reduce the global spread of COVID-19. Ferguson et al. (2020) indicated that home quarantine, social distancing of the entire population, and closure of schools and universities can reduce transmission of the virus. However, another study indicated that the key to the implementation of this strategy is that residents voluntarily comply with home quarantine requirements (Pan et al., 2020). In reality, some residents are still inattentive to the home quarantine strategy and do not comply with the requirements, which vastly reduces the effectiveness of containment strategies and indirectly contributes to the spread of the epidemic.

In an infectious disease pandemic, there are different motivations for residents' adherence to recommendations about social distancing (e.g., desire to protect self and others), and external circumstances or motivators (e.g., work/school conducted remotely) contribute to engagement in and adherence to preventative behaviors, such as social distancing (Guo et al., 2020). These motivations also likely interact with various sociodemographic variables, such as gender, age, socioeconomic and health status, and household size and composition (Guo et al., 2020). There are multiple factors influencing residents' willingness to comply with home quarantine according to previous studies (Webster et al., 2020). For instance, a potential factor is the objective condition of the individual based on income and employment status and the individual's state of health (Cava et al., 2005; Porten et al., 2006; Bodas and Peleg, 2020). Another factor is the subjective psychological status of residents, which reflects their subjective cognitive situation and degree of panic in this crisis (Cui et al., 2020). The third is the environmental factors of residents, which include the government's response to the crisis and residents' satisfaction with the government's actions (Hsu et al., 2006; Desclaux et al., 2017).

Several studies conducted during the COVID-19 pandemic have explored the willingness of residents to comply with quarantine measures and the influencing factors. However, some studies considered only the impact of demographic and social characteristics and economic factors on residents and failed to fully consider the role of psychological status (Geldsetzer, 2020; Kamenidou et al., 2020). There were also studies that considered the role of mental health and risk perception but did not use professional scales to measure the psychological condition of the residents (Li et al., 2020; Roy et al., 2020; Atchison et al., 2021), such as the Center for Epidemiological Studies Depression Scale (CES-D), the 10-item Connor-Davidson Resilience Scale (CD-RISC 10), and the Perceived Social Support Scale (PSSS). Therefore, it is necessary to conduct research based on full consideration of the various potential factors and the use of specialized measurement tools.

Various models of health behavior change conceptualize motivation as a central predictor for the adoption and maintenance of preventative health behaviors. For example, the capability-opportunity-motivation-behavior (COM-B) model (Michie et al., 2011) posits that the interaction between individual capability (or having the necessary knowledge and skills) and opportunity (physical, social, and environmental support) directly influences motivation to engage in a behavior (reflective and automatic processes driving behavior), which leads to behavior change and maintenance. Self-determination theory (Ryan and Deci, 2000) suggests that there are two types of motivations that drive behavior change: intrinsic motivation, where the individual derives pleasure from the behavior, and extrinsic motivation, where external pressures facilitate adherence to behavior. However, few studies have focused on the influencing factors of home quarantine based on these theories and the influencing factors proposed by previous studies. Therefore, we investigated the willingness of residents to quarantine at home and explored the main factors, which has significance for the improvement and implementation of the home quarantine strategy in future global public health emergency response.

Given that the home quarantine strategy entails significant lifestyle changes for the general population and may potentially be required for months or years to come (Zhang et al., 2020), it is important to understand what facilitates or prevents adherence to these measures so that public health interventions can be developed in a timely manner. Because most countries have relaxed their social and physical distancing measures compared to the measures taken in the early days of the epidemic, it is crucially important to determine the factors that might affect adherence to these preventive health behaviors in the long run.

## Methods

### Ethics Statement

This study protocol was approved by the institutional review board of Tongji Medical College of Huazhong University of Science and Technology, Wuhan, China (IORG No: IORG0003571).

### Study Participants

We conducted a cross-sectional survey in China from January 31 to February 29, 2020. We stratified the respondents mainly according to geographical area: the eastern, central, and western regions of China. We adopted a convenience sampling strategy to recruit participants. A total of 3,495 residents received the questionnaire. The response rate was 97.2%, and 3,398 complete questionnaires were employed for the results analysis.

### Survey Tools

The questionnaire consisted of five sections: sociodemographic information of the respondents, COVID-19-related cognitive evaluation and protective behaviors, the Center for Epidemiological Studies-Depression Scale (CES-D), the 10-item Connor-Davidson Resilience Scale (CD-RISC 10), and the Perceived Social Support Scale (PSSS). Table 1 presents the scales-items used, where they were adopted from, and validity and reliability measures.

**Table 1:** Details of the Center for Epidemiological Studies-Depression Scale (CES-D), 10-item Connor-Davidson Resilience Scale (CD-RISC 10), and Perceived Social Support Scale (PSSS).

**Scale**	**Items**	**Each item ranged**	**Construct**	**Average Variance Extracted (AVE)**	**Composite Reliability (CR)**	**Cronbach α**	**Application in Chinese population**
Center for Epidemiologic Studies Depression Scale (CES-D)	20	0–3	Full scale Depressed mood Guilt and unworthiness Helplessness and hopelessness Psychomotor hysteresis Loss of appetite Sleep disorders	0.7 0.7 0.6 0.6 0.8 0.7 0.7	0.8 0.7 0.8 0.8 0.9 0.7 0.7	0.9 0.9 0.8 0.8 0.9 0.9 0.8	Chen et al., 2015; Yang et al., 2015; Wang et al., 2019a,b
10-item Connor-Davidson Resilience Scale (CD-RISC 10)	10	0–4	Full scale Change Personal problems Disease Pressure The feeling of failure and pain	0.8 0.8 0.8 0.7 0.7 0.7	0.7 0.7 0.6 0.7 0.7 0.7	0.8 0.7 0.7 0.8 0.8 0.8	Li et al., 2016; Meng et al., 2019; Cheng et al., 2020
Perceived Social Support Scale (PSSS)	12	1–7	Full scale Family support Friends support Other support	0.8 0.7 0.8 0.8	0.8 0.7 0.7 0.8	0.8 0.8 0.8 0.9	Liu et al., 2016; Xu et al., 2019; Xiao et al., 2020

#### Center for Epidemiologic Studies Depression Scale (CES-D)

The CES-D is a 20-item self-rating scale for the measurement of depressive symptoms (Adams et al., 2019). It was designed for use in the general population and has been successfully employed to assess depression in a variety of community samples. The categorical response variables for each item ranged from 0 (Rarely or none of the time) to 3 (Most or all of the time). Higher scores reflected lower levels of positive emotional well-being. The Cronbach's alpha coefficient of this scale was 0.9 in this study.

#### 10-Item Connor-Davidson Resilience Scale (CD-RISC 10)

The CD-RISC 10 was developed by Campbell-Sills and Stein as a viable alternative to the original scale after a review of its efficacy revealed an unstable factor structure. The revised scale is a self-report measure with excellent psychometric properties and has generally been shown to be unidimensional in nature (Siddaway et al., 2017). The CD-RISC10 was used to measure the psychological resilience of residents (Cosco et al., 2016). This 10-item scale uses a response scale from 0 (never or not possible) to 4 (always or extremely possible) (Reid, 2016). The total score of the psychological resilience scale is the sum of the scores of the items and ranges from 0 to 40 points. Higher scores indicate higher psychological resilience (Cosco et al., 2016). The Cronbach's alpha coefficient of this scale was 0.8 in this study.

#### Perceived Social Support Scale (PSSS)

Another measure used was the perceived social support status among Chinese community dwellers during the COVID-19 pandemic. The Perceived Social Support Scale (PSSS) has 12 items in three dimensions: family support, friend support, and significant other support (Kuru and Piyal, 2018). It has a three-factor structure, with each subscale comprising four items addressing practical help, emotional support, and availability to discuss problems and help in decision making. These 12 items were assessed on a seven-point Likert scale ranging from 1 (strongly disagree) to 7 (strongly agree). The total scores ranged from 12 to 84, with higher scores indicating better social support among residents. Scores of 12–36 indicate low social support; 37–60 indicate moderate social support; and 61–84 indicate high social support (Li et al., 2017). The Cronbach's alpha coefficient of this scale was 0.8 in this study.

### Data Collection and Quality Control

The design of the questionnaire was based on a literature review, small group discussion, and simulated interviews (Li et al., 2019). We invited experts for group discussion to improve the professionalism of the questionnaire based on the Delphi method. In addition, we conducted a pilot study to ensure that the language of the questionnaire could be understood and accepted by most residents. Next, we leveraged WeChat (China's largest messaging platform with nearly 1 billion users, similar to WhatsApp in Western countries) to send a hyperlink of the online questionnaire, which was designed using “Survey Star (wjx.cn),” to participants. The researcher entered the data into the Internet database to ensure accuracy (Yu et al., 2019).

### Data Analysis

The dependent variable is whether the home quarantine strategy was implemented. In the multivariable model, predictive variables included region, dwelling place, age, sex, marital status, education level, income level, physical condition, drinking habits, smoking status, chronic disease status, whether self or relative had been diagnosed with COVID-19, CES-D score, and CD-RISC 10 score (Mancilla-Galindo et al., 2020; Simpson et al., 2020).

Descriptive analysis was conducted to determine the characteristics of the participants, including the quantity and percentage. No clustering was observed in the respondents (correlation = 0.03, *p* < 0.001). We used stepwise multivariable logistic regression analysis to determine the predictors of home quarantine, i.e., level for selection and elimination: *p* = 0.05 and *p* = 0.10, respectively (Wang et al., 2020). We performed analyses by using SAS version 9.2 (SAS Institute, Cary, NC), and all tests were 2-sided with a significance level of 0.05 (Yu et al., 2018).

## Results

Table 2 reports the sociodemographic characteristics of the 3,398 respondents. The mean age was 27.6 years (*SD* = 7.7), and the majority of respondents were female (66.5%). Among the respondents, 585 (17.2%), 2,716 (69.0%), and 637 (18.8%) were from eastern, central, and western China, respectively. Most respondents (95.6%) were Han Chinese and single (68.9%). Approximately 62.0% of respondents were students, soldiers, or freelancers. More than half of the respondents (58.2%) lived in urban areas, and more than half of the respondents (53.7%) had a low mean monthly family income. Most respondents (86.2%) had a good health status and medical insurance (86.6%). A total of 2,936 (86.4%) respondents carried out home quarantine. The CES-D, CD-RISC 10, and PSSS results across respondents and items showed that the average scores for community residents' social support were 28.4 (*SD* = 10.6), 27.1 (*SD* = 8.0), and 66.1 (*SD* = 12.3), respectively.

**Table 2:** Statistical description of study samples.

**Variables**	***N* (%)**
Total	3 398 (100)
Gender	
Male	1,138 (33.5)
Female	2 260 (66.5)
Age group, y	
18–44	2,994 (88.1)
45–59	367 (10.8)
>60	37 (1.1)
Region	
Eastern China	585 (17.2)
Central China	2,716 (69.0)
Western China	637 (18.8)
Ethnicity	
Han Chinese	3,248 (95.6)
Minorities	150 (4.4)
Marital status	
Single/widow/divorced	2,340 (68.9)
Married	1,058 (31.1)
Place of residence	
Urban	1,976 (58.2)
Rural	1,422 (41.9)
Highest educational level	
Primary school or below	16 (0.5)
Junior middle school	79 (2.3)
Senior middle school	291 (8.6)
College degree or above	3,012 (88.6)
Employment status	
Employed	1,220 (35.9)
Retired	41 (1.2)
Unemployed	28 (0.8)
Others (students, soldiers, freelancers)	2109 (62.1)
Mean monthly family income	
Higher	203 (6.0)
Middle	1,369 (40.3)
Lower	1,826 (53.7)
Medical insurance status	
Present	2,941 (86.6)
Absent	457 (13.5)
Health status	
Good	2,930 (86.2)
Fair	439 (12.9)
Poor	29 (0.9)
Smoking status	
Current smoker	192 (5.7)
Ex-smoker	85 (2.5)
Non-smoker	3,121 (91.9)
Alcohol consumption status	
Current drinker	397 (11.7)
Ex-drinker	73 (2.2)
Abstainer	2,928 (86.2)
Chronic disease status	
Present	289 (8.5)
Absent	3109 (91.5)
Home quarantine was carried out	
Yes	2,936 (86.4)
No	462 (13.6)
Patients and relatives diagnosed with COVID-19	
Yes	17 (0.5)
No	3,381 (99.5)
Awareness of primary symptoms of COVID-19	
Aware	3,010 (88.6)
Fair	346 (10.2)
Unaware	42 (1.2)
Level of concern for COVID-19	
Concerned	2,995 (88.1)
Moderate	356 (10.5)
Not concerned	47 (1.4)
Willingness to accept recommendations on relevant protective measures	
(such as wearing masks or not participating in gatherings)	
Willing	3,149 (92.7)
Fair	226 (6.7)
Unwilling	23 (0.7)
Understanding of local quarantine measures	
Understood	2,795 (82.3)
Fair	458 (13.5)
Not understood	145 (4.3)

Table 3 shows the odds ratio (OR), confidence interval (CI), and significance (p) values from the multivariable analyses. Respondents who were male (*OR* = 1.5, 95% CI: 1.3 ~ 1.9), lived in western and central China (*OR* = 1.6, 95% CI: 1.2 ~ 2.0), were aware of the primary symptoms of COVID-19 (*OR* = 1.7, 95% CI: 1.3 ~ 2.1), were willing to accept recommendations on relevant protective measures (*OR* = 1.1, 95% CI: 1.0 ~ 1.2), understood local quarantine measures (*OR* = 1.1, 95% CI: 1.1 ~ 1.2), had better resilience (*OR* = 1.4, 95% CI: 1.1 ~ 2.0), and had better social support (*OR* = 1.3, 95% CI: 1.1 ~ 1.4) were more likely to implement home quarantine. Respondents who were married (*OR* = 0.5, 95% CI: 0.4 ~ 0.7), were employed (*OR* = 0.8, 95% CI: 0.7 ~ 0.9), were healthy (*OR* = 0.8, 95% CI: 0.7 ~ 0.9, and had high depression scores (*OR* = 0.4, 95% CI: 0.3 ~ 0.5) were more likely to refuse to implement home quarantine.

**Table 3:** Results of the multivariable analyses for home quarantine among the Chinese residents.

**Variables**	**Odds Ratio (OR)**	**95% Confidence Interval (CI)**	**Significance (P)**
Gender (Reference: Female)	1.5	1.3 ~ 1.9	0.009
Region (Reference: Eastern China)	1.6	1.2~ 2.0	<0.001
Marital status (Reference: Single/widow/divorced)	0.5	0.4 ~ 0.7	0.005
Employment status (Reference: Others [students, soldiers, freelancers])	0.8	0.7 ~ 0.9	0.009
Health status (Reference: Poor)	0.8	0.7 ~ 0.9	0.006
Awareness of primary symptoms of COVID-19 (Reference: Unaware)	1.7	1.3 ~ 2.1	0.009
Willingness to accept recommendations on relevant protective measures (such as wearing masks or not participating in gatherings) (Reference: Unwilling)	1.1	1.1 ~ 1.2	0.004
Understanding of local quarantine measures (Reference: Not understood)	1.1	1.1 ~ 1.2	0.009
Depression condition	0.4	0.3 ~ 0.5	<0.001
Psychological resilience condition	1.4	1.1 ~ 2.0	<0.001
Perceived social support condition	1.3	1.1 ~ 1.4	<0.001

## Discussion

In the face of the outbreak and rapid spread of COVID-19, China actively adopted a series of effective non-pharmaceutical intervention measures (West et al., 2020). In particular, China implemented the home quarantine strategy of “Reduce travel and contact with others” nationwide, which provided an important boost to COVID-19 prevention and control. This large population-based cross-sectional survey showed that most respondents (86.40%) carried out home quarantine, and whether residents complied with home quarantine had a significant relationship with their gender, region, marital status, employment status, health status, awareness of the primary symptoms of COVID-19, willingness to accept recommendations on relevant protective measures, understanding of local quarantine measures, depression status, resilience, and perceived social support.

Males were more likely to comply with home quarantine, and females were less likely to comply with the strategy. This may reflect the fact that women are more likely to do outside activities. Married residents were more likely to refuse to comply with home quarantine than single/divorced/widowed residents. This may be due to the fact that families can provide some support to individuals during an emergency, so residents with spouses are more confident about overcoming the crisis (Mediouni et al., 2020). This phenomenon also appeared for the region factor. Residents living in eastern China (where economic conditions are better than those in central and western China) had a more relaxed attitude toward the epidemic and were less inclined to implement the home quarantine strategy. One possible explanation is that residents living in eastern China experience better economic conditions, and the living standards are generally higher there than in the central and western regions. Affected by the economic level, residents in different regions showed different mental health responses in the face of the COVID-19 outbreak. However, better mental health may cause such people to lose their alertness to risk. This indicates that countries worldwide should improve the level of economic development and urbanization and simultaneously strengthen public awareness and implementation of policy (Chevance et al., 2020).

The health status of residents was also an important factor affecting the implementation of the home quarantine strategy. Residents with poor health were more likely to implement the home quarantine strategy than those with good health. This may be because residents in poorer health were suffering from psychological stress and were more alert to the risk of illness. Therefore, countries worldwide should actively carry out the detection of suspected patients and strengthen care for residents with poor health (Wang et al., 2020). The more residents knew about the underlying symptoms of COVID-19, the more likely they were to implement a home quarantine strategy. A better understanding of the underlying symptoms of COVID-19 reflected the greater concern of these residents for their own health. Similarly, the more residents understood local government strategies, the more willing they were to implement home quarantine, indicating that a government's policies can provide psychological support to the public. In the face of the COVID-19 outbreak, the government could take effective measures to treat confirmed cases and control the spread of the epidemic, and the public will be more confident in facing the crisis (Reynolds et al., 2020). Thus, countries should respond to people's concerns in a timely manner and take measures to safeguard people's well-being and protect their physical and mental health (Shi et al., 2020). People who were more depressed and had lower levels of mental resilience and social support were more willing to implement the home quarantine strategy. This suggests that psychological factors play an important role in the implementation of this strategy. Some residents will have a poor psychological state in the face of emergencies, and they may better implement national policies. However, there are also some residents who are more optimistic and in a better psychological state, and these residents are more likely to reject national policies (Wang et al., 2020). Countries should actively carry out health education and policy popularization to encourage residents to maintain a good psychological state while improving the implementation of policies.

China is a growing developing country and has a large rural population (Tan et al., 2018). Although China has made great efforts to develop medical and health services and its emergency response to public health emergencies since the SARS epidemic in 2003, it still had to take strong measures and pay a heavy price to contain the spread of COVID-19 in the face of national transmission (Zheng, 2020). COVID-19 is currently raging around the world, making it the worst global pandemic of this century. In the face of the COVID-19 outbreak, how countries promote the implementation of the home quarantine strategy has become a very important international topic. Governments should pay close attention to the policy implementation and psychological status of residents while actively implementing home quarantine strategies. In the long run, countries should further improve their economies, urbanization, and resilience while actively treating confirmed cases, isolating vulnerable populations, strengthening health education, and improving residents' understanding of health emergency policies to weather future global epidemics.

This study has several limitations. In the questionnaire, some potential predictors of the implementation of the home quarantine strategy among Chinese residents were not investigated, such as cultural factors. In addition, this is a cross-sectional study, which limits the establishment of temporal and causal relationships. However, this study has several advantages. First, this is the first national study on the prevalence and risk factors of home quarantine strategy implementation among residents. Second, with the popularization of smartphones and rapid development of communications tools, an Internet-based survey method could be employed. This study was conducted on an advanced interaction platform, and a higher response rate was obtained by chatting with the survey subjects. The academic contribution of this study is that it considered the potential factors that have been reported by previous studies to influence residents' willingness to implement the home quarantine strategy, especially the influence of psychological factors. In addition, standardized scales were used in the investigation, including the Center for Epidemiological Studies-Depression Scale (CES-D), 10-item Connor-Davidson resilience scale (CD-RISC 10), and Perceived Social Support Scale (PSSS), which enhanced the accuracy of the research conclusions.

Future research could explore more factors that may influence residents' willingness based on this study, such as culture, ethnicity, or religion. In addition, longitudinal studies should be conducted in the future to evaluate the relationship between various influencing factors and the prevalence and risk factors of home quarantine among residents. Countries worldwide are still facing the threat of COVID-19, and the implementation of home quarantine should be taken seriously by governments. Our findings indicate that attention and action are needed to promote home quarantine strategy enforcement. Therefore, global policymakers should take appropriate measures to improve the implementation of the home quarantine strategy among residents, including executing active treatment, strengthening health education, improving residents' awareness of health emergency policies in the short term, and improving the economic level, health, and resilience of the population in the long run.

## Conclusions

The home quarantine strategy is an important strategy for dealing with COVID-19 and was first implemented in China and later promoted in many countries around the world. However, whether this policy can be widely implemented is related to the residents' material foundation, mental condition, and living environment. The investigation revealed that gender, region, marital status, employment status, health status, awareness of the primary symptoms of COVID-19, willingness to accept recommendations on relevant protective measures, understanding of local quarantine measures, depression, psychological resilience, and perceived social support were the important factors affecting the implementation of the home quarantine strategy. This study provides a solid reference for global home quarantine policymakers as well as lessons for dealing with future outbreaks.

## Data Availability Statement

The original contributions presented in the study are included in the article/supplementary material, further inquiries can be directed to the corresponding author.

## Ethics Statement

This study protocol was approved by the Institutional Review Board of Tongji Medical College of Huazhong University of Science and Technology, Wuhan, China (IORG No: IORG0003571). The patients/participants provided their written informed consent to participate in this study.

## Author Contributions

LL, XS, and YG conceived and designed the study. JF, ZLe, and KT participated in the acquisition of data. XZ and HC analyzed the data. JL and YW gave advice on methodology. LL and XS drafted the manuscript. ZLu and YG revised the manuscript. YG is the guarantor of this work and had full access to all the data in the study and takes responsibility for its integrity and the accuracy of the data analysis. All authors read and approved the final manuscript.

## Funding

This study was supported by the Fundamental Research Funds for the Central Universities (2020kfyXJJS059) and Guizhou Province Science and Technology Support Project ([2020]4Y165).

## Conflict of Interest

The authors declare that the research was conducted in the absence of any commercial or financial relationships that could be construed as a potential conflict of interest.

## Publisher's Note

All claims expressed in this article are solely those of the authors and do not necessarily represent those of their affiliated organizations, or those of the publisher, the editors and the reviewers. Any product that may be evaluated in this article, or claim that may be made by its manufacturer, is not guaranteed or endorsed by the publisher.

## References

[B1] Adams L. B. Gottfredson N. Lightfoot A. F. Corbie-Smith G. Golin C. Powell W. (2019). Factor analysis of the CES-D 12 among a community sample of black men. Am. J. Mens Health 13:1557988319834105. 10.1177/1557988319834105 30894043 PMC6440056

[B2] Atchison C. Bowman L. R. Vrinten C. Redd R. Pristerà P. Eaton J.. (2021). Early perceptions and behavioural responses during the COVID-19 pandemic: a cross-sectional survey of UK adults. BMJ Open 11:e043577. 10.1136/bmjopen-2020-043577 33397669 PMC7783373

[B3] Bauch C. T. Anand M. (2020). COVID-19: when should quarantine be enforced? Lancet Infect. Dis. 20, 994–995. 10.1016/S1473-3099(20)30428-X 32445711 PMC7239632

[B4] Bodas M. Peleg K. (2020). Income assurances are a crucial factor in determining public compliance with self-isolation regulations during the COVID-19 outbreak - cohort study in Israel. Isr. J. Health Policy Res. 9:54. 10.1186/s13584-020-00418-w 33081833 PMC7573868

[B5] Cava M. A. Fay K. E. Beanlands H. J. McCay E. A. Wignall R. (2005). Risk perception and compliance with quarantine during the SARS outbreak. J. Nurs. Scholarsh. 37, 343–347. 10.1111/j.1547-5069.2005.00059.x 16396407

[B6] Chen J. Chen S. Landry P. F. (2015). Urbanization and mental health in China: linking the 2010 population census with a cross-sectional survey. Int. J. Environ. Res. Public Health 12, 9012–2904. 10.3390/ijerph120809012 26264013 PMC4555260

[B7] Cheng C. Dong D. He J. Zhong X. Yao S. (2020). Psychometric properties of the 10-item Connor-Davidson Resilience Scale (CD-RISC-10) in Chinese undergraduates and depressive patients. J. Affect. Disord. 261, 211–220. 10.1016/j.jad.2019.10.018 31654919

[B8] Chevance A. Gourion D. Hoertel N. Llorca P. M. Thomas P. Bocher R.. (2020). Ensuring mental health care during the SARS-CoV-2 epidemic in France: a narrative review. Encephale 46, 193–201. 10.1016/j.encep.2020.04.005 32370982 PMC7174154

[B9] Cosco T. D. Kaushal A. Richards M. Kuh D. Stafford M. (2016). Resilience measurement in later life: a systematic review and psychometric analysis. Health Qual. Life Outcomes 14:16. 10.1186/s12955-016-0418-6 26821587 PMC4730639

[B10] Cui T. Yang G. Ji L. Zhu L. Zhen S. Shi N.. (2020). Chinese residents' perceptions of COVID-19 during the pandemic: online cross-sectional survey study. J. Med. Internet Res. 22:e21672. 10.2196/21672 33152684 PMC7690970

[B11] Desclaux A. Badji D. Ndione A. G. Sow K. (2017). Accepted monitoring or endured quarantine? Ebola contacts' perceptions in Senegal. Soc. Sci. Med. 178, 38–45. 10.1016/j.socscimed.2017.02.009 28192745

[B12] Ferguson N. M. Laydon D Nedjati-Gilani G. Imai N. Ainslie K. Baguelin M.. (2020). Impact of Non-Pharmaceutical Interventions (NPIs) to Reduce COVID-19 Mortality and Healthcare Demand. London: Imperial College COVID-19 Response Team.

[B13] Geldsetzer P. (2020). Use of rapid online surveys to assess people's perceptions during infectious disease outbreaks: a cross-sectional survey on COVID-19. J. Med. Internet Res. 22:e18790. 10.2196/18790 32240094 PMC7124956

[B14] Guo Y. Cheng C. Zeng Y. Li Y. Zhu M. Yang W.. (2020). Mental health disorders and associated risk factors in quarantined adults during the COVID-19 outbreak in China: cross-sectional study. J. Med. Internet Res. 22:e20328. 10.2196/20328 32716899 PMC7419152

[B15] Hsu C. C. Chen T. Chang M. Chang Y. K. (2006). Confidence in controlling a SARS outbreak: experiences of public health nurses in managing home quarantine measures in Taiwan. Am. J. Infect. Control 34, 176–181. 10.1016/j.ajic.2005.11.008 16679173 PMC7115257

[B16] Kamenidou I. E. Stavrianea A. Liava C. (2020). Achieving a Covid-19 free country: citizens preventive measures and communication pathways. Int J Environ Res Public Health 17. 10.3390/ijerph17134633 32605097 PMC7370045

[B17] Kuru N. Piyal B. (2018). Perceived social support and quality of life of parents of children with Autism. Niger. J. Clin. Pract. 21, 1182–1189. 10.4103/njcp.njcp_13_18 30156205

[B18] Li G. Kong L. Zhou H. Kang X. Fang Y. Li P. (2016). Relationship between prenatal maternal stress and sleep quality in Chinese pregnant women: the mediation effect of resilience. Sleep Med. 25, 8–12. 10.1016/j.sleep.2016.02.015 27823722

[B19] Li W. Yang Y. Liu Z. H. Zhao Y. J. Zhang Q. Zhang L.. (2020). Progression of mental health services during the COVID-19 outbreak in China. Int. J. Biol. Sci. 16, 1732–1738. 10.7150/ijbs.45120 32226291 PMC7098037

[B20] Li X. Li T. Chen J. Xie Y. An X. Lv Y.. (2019). A WeChat-based self-management intervention for community middle-aged and elderly adults with hypertension in guangzhou, china: a cluster-randomized controlled trial. Int. J. Environ. Res. Public Health 16:4058. 10.3390/ijerph16214058 31652688 PMC6862068

[B21] Li Y. Long Z. Cao D. Cao F. (2017). Social support and depression across the perinatal period: a longitudinal study. J. Clin. Nurs. 26, 2776–2783. 10.1111/jocn.13817 28334472

[B22] Liu L. Gou Z. Zuo J. (2016). Social support mediates loneliness and depression in elderly people. J. Health Psychol. 21, 750–758. 10.1177/1359105314536941 24925547

[B23] Mancilla-Galindo J. Vera-Zertuche J. M. Navarro-Cruz A. R. Segura-Badilla O. Reyes-Velázquez G. Tepepa-López F. J.. (2020). Development and validation of the patient history COVID-19 (PH-Covid19) scoring system: a multivariable prediction model of death in Mexican patients with COVID-19. Epidemiol. Infect. 148:e286. 10.1017/S0950268820002903 33239114 PMC7729170

[B24] Matias T. Dominski F. H. Marks D. F. (2020). Human needs in COVID-19 isolation. J. Health Psychol. 25, 871–882. 10.1177/1359105320925149 32375564

[B25] McCloskey B. Zumla A. Ippolito G. Blumberg L. Arbon P. Cicero A.. (2020). Mass gathering events and reducing further global spread of COVID-19: a political and public health dilemma. Lancet 395, 1096–1099. 10.1016/S0140-6736(20)30681-4 32203693 PMC7138150

[B26] Mediouni M. Madiouni R. Kaczor-Urbanowicz K. E. (2020). COVID-19: How the quarantine could lead to the depreobesity. Obes. Med. 19:100255. 10.1016/j.obmed.2020.100255 32427138 PMC7227567

[B27] Meng M. He J. Guan Y. Zhao H. Yi J. Yao S.. (2019). Factorial Invariance of the 10-Item connor-davidson resilience scale across gender among Chinese elders. Front. Psychol. 10:1237. 10.3389/fpsyg.2019.01237 31214071 PMC6554443

[B28] Michie S. van Stralen M. M. West R. (2011). The behaviour change wheel: a new method for characterising and designing behaviour change interventions. Implement. Sci. 6:42. 10.1186/1748-5908-6-42 21513547 PMC3096582

[B29] Pan A. Liu L. Wang C. Guo H. Hao X. Wang Q.. (2020). Association of public health interventions with the epidemiology of the COVID-19 outbreak in Wuhan, China. JAMA 323, 1915–1923. 10.1001/jama.2020.6130 32275295 PMC7149375

[B30] Porten K. Faensen D. Krause G. (2006). SARS outbreak in Germany 2003: workload of local health departments and their compliance in quarantine measures–implications for outbreak modeling and surge capacity? J. Public Health Manag. Pract. 12, 242–247. 10.1097/00124784-200605000-00004 16614559

[B31] Reid R. (2016). Psychological resilience. Med. Leg. J. 84, 172–184. 10.1177/0025817216638781 27030506

[B32] Reynolds J. P. Stautz K. Pilling M. van der Linden S. Marteau T. M. (2020). Communicating the effectiveness and ineffectiveness of government policies and their impact on public support: a systematic review with meta-analysis. R. Soc. Open Sci. 7:190522. 10.1098/rsos.190522 32218927 PMC7029938

[B33] Roy D. Tripathy S. Kar S. K. Sharma N. Verma S. K. Kaushal V. (2020). Study of knowledge, attitude, anxiety & perceived mental healthcare need in Indian population during COVID-19 pandemic. Asian J. Psychiatr. 51:102083. 10.1016/j.ajp.2020.102083 32283510 PMC7139237

[B34] Ryan R. M. Deci E. L. (2000). Self-determination theory and the facilitation of intrinsic motivation, social development, and well-being. Am. Psychol. 55, 68–78. 10.1037/0003-066X.55.1.68 11392867

[B35] Shi L. Lu Z. A. Que J. Y. Huang X. L. Liu L. Ran M. S.. (2020). Prevalence of and risk factors associated with mental health symptoms among the general population in china during the Coronavirus Disease 2019 pandemic. JAMA Netw. Open 3:e2014053. 10.1001/jamanetworkopen.2020.14053 32609353 PMC7330717

[B36] Siddaway A. P. Wood A. M. Taylor P. J. (2017). The Center for Epidemiologic Studies-Depression (CES-D) scale measures a continuum from well-being to depression: testing two key predictions of positive clinical psychology. J. Affect. Disord. 213, 180–186. 10.1016/j.jad.2017.02.015 28254608 PMC6191531

[B37] Simpson N. B. Shankar-Hari M. Rowan K. M. Cecconi M. von Dadelszen P. Huning E. Y.. (2020). Maternal risk modeling in critical care-development of a multivariable risk prediction model for death and prolonged intensive care. Crit. Care Med. 48, 663–672. 10.1097/CCM.0000000000004223 31923028

[B38] Tan X. Wu Q. Shao H. (2018). Global commitments and China's endeavors to promote health and achieve sustainable development goals. J. Health Popul. Nutr. 37:8. 10.1186/s41043-018-0139-z 29650054 PMC5898031

[B39] Wang J. Liao Y. Wang X. Li Y. Jiang D. He J.. (2020). Incidence of novel coronavirus (2019-nCoV) infection among people under home quarantine in Shenzhen, China. Travel Med. Infect. Dis. 37:101660. 10.1016/j.tmaid.2020.101660 32247931 PMC7270728

[B40] Wang J. Wang Z. (2020). Strengths, Weaknesses, Opportunities and Threats (SWOT) analysis of China's prevention and control strategy for the COVID-19 epidemic. Int. J. Environ. Res. Public Health 17:2235. 10.3390/ijerph17072235 32225019 PMC7178153

[B41] Wang R. Bishwajit G. Zhou Y. Wu X. Feng D. Tang S.. (2019a). Intensity, frequency, duration, and volume of physical activity and its association with risk of depression in middle- and older-aged Chinese: evidence from the China health and retirement longitudinal study, 2015. PLoS ONE 14:e0221430. 10.1371/journal.pone.0221430 31425559 PMC6699736

[B42] Wang R. Liu Y. Xue D. Helbich M. (2019b). Depressive symptoms among Chinese residents: how are the natural, built, and social environments correlated? BMC Public Health 19:887. 10.1186/s12889-019-7171-9 31277619 PMC6611031

[B43] Webster R. K. Brooks S. K. Smith L. E. Woodland L. Wessely S. Rubin G. J. (2020). How to improve adherence with quarantine: rapid review of the evidence. Public Health 182, 163–169. 10.1016/j.puhe.2020.03.007 32334182 PMC7194967

[B44] West R. Michie S. Rubin G. J. Amlôt R. (2020). Applying principles of behaviour change to reduce SARS-CoV-2 transmission. Nat. Hum. Behav. 4, 451–459. 10.1038/s41562-020-0887-9 32377018

[B45] Xiao H. Zhang Y. Kong D. Li S. Yang N. (2020). The effects of social support on sleep quality of medical staff treating patients with Coronavirus Disease 2019 (COVID-19) in January and February 2020 in China. Med. Sci. Monit. 26:e923549. 10.12659/MSM.923549 32132521 PMC7075079

[B46] Xu Q. Li S. Yang L. (2019). Perceived social support and mental health for college students in mainland China: the mediating effects of self-concept. Psychol. Health Med. 24, 595–604. 10.1080/13548506.2018.1549744 30451537

[B47] Yang L. Jia C. X. Qin P. (2015). Reliability and validity of the Center for Epidemiologic Studies Depression Scale (CES-D) among suicide attempters and comparison residents in rural China. BMC Psychiatry 15:76. 10.1186/s12888-015-0458-1 25886490 PMC4426543

[B48] Yu P. F. Liu J. T. Ma Z. J. Zhong M. Li X. C. Jiang H. (2018). [Logistic regression analysis on the outcome predictive factors of ruptured lumbar disc herniation]. Zhongguo Gu Shang 31, 522–527. 10.3969/j.issn.1003-0034.2018.06.008 29945407

[B49] Yu Q. Xu L. Li L. Zhi M. Gu Y. Wang X.. (2019). Internet and WeChat used by patients with Crohn's disease in China: a multi-center questionnaire survey. BMC Gastroenterol. 19:97. 10.1186/s12876-019-1011-3 31221086 PMC6584988

[B50] Zhang X. M. Zhou H. E. Zhang W. W. Dou Q. L. Li Y. Wei J.. (2020). Assessment of Coronavirus Disease 2019 community containment strategies in Shenzhen, China. JAMA Netw. Open 3:e2012934. 10.1001/jamanetworkopen.2020.12934 32568401 PMC7309437

[B51] Zheng J. (2020). SARS-CoV-2: an emerging coronavirus that causes a global threat. Int. J. Biol. Sci. 16, 1678–1685. 10.7150/ijbs.45053 32226285 PMC7098030

